# Mycoprotein: environmental impact and health aspects

**DOI:** 10.1007/s11274-019-2723-9

**Published:** 2019-09-23

**Authors:** Pedro F. Souza Filho, Dan Andersson, Jorge A. Ferreira, Mohammad J. Taherzadeh

**Affiliations:** 1 Swedish Centre for Resource Recovery, University of Borås, Borås, Sweden; 2 Faculty of Caring Science, Work Life and Social Welfare, University of Borås, Borås, Sweden; 3 Present Address: Laboratory of Biochemical Engineering, Chemical Engineering Department, Federal University of Rio Grande do Norte, Natal, 59078-970 Brazil

**Keywords:** Alternative protein, Amino acids, Human health, Life cycle analysis, Meat substitute, Mycoprotein

## Abstract

The term mycoprotein refers to the protein-rich food made of filamentous fungal biomass that can be consumed as an alternative to meat. In this paper, the impact caused by the substitution of animal-origin meat in the human diet for mycoprotein on the health and the environment is reviewed. Presently, mycoprotein can be found in the supermarkets of developed countries in several forms (e.g. sausages and patties). Expansion to other markets depends on the reduction of the costs. Although scarce, the results of life cycle analyses of mycoprotein agree that this meat substitute causes an environmental impact similar to chicken and pork. In this context, the use of inexpensive agro-industrial residues as substrate for mycoprotein production has been investigated. This strategy is believed to reduce the costs involved in the fungal cultivation and lower the environmental impact of both the mycoprotein and the food industry. Moreover, several positive effects in health have been associated with the substitution of meat for mycoprotein, including improvements in blood cholesterol concentration and glycemic response. Mycoprotein has found a place in the market, but questions regarding the consumer’s experience on the sensory and health aspects are still being investigated.

## Introduction

During the past 60 years, the global agricultural production has been thoroughly improved to meet the demands of a rapidly growing population. With an increase of just 10% in the amount of agricultural land used, the global food production doubled (FAO 2010). This strategy, however, together with changes in the lifestyle, poverty, population pressures, and urbanization, has deeply affected the human diet and health (Augustin et al. 2016).

The food sector constitutes one of the largest contributors to both local and global environmental impact and resource use. Several studies associate losses of biodiversity and degradation of ecosystems with food production (Dunne et al. 2002; Röös et al. 2013; Tscharntke et al. 2012). It is estimated that between 70 and 85% of the water footprint caused by human activities is associated with the agricultural activity (Smetana et al. 2015). Moreover, more than 30% of the total greenhouse gases (GHG) emitted by humans are a side product of the agricultural sector, with more than half of it (approximately 18%) being connected with the production of meat (Smetana et al. 2015; Steinfeld et al. 2006).

The global population is estimated to decelerate its growth, reaching a plateau at around 9 billion people near the middle of this century; providing food to this population will add extra pressure to the food system (Godfray et al. 2010). These facts are regarded as challenges to the future of the food and nutrition security, and led to the proposition of new food systems to improve public health. The propositions are based on the concept of a sustainable diet, i.e., a diet with reduced environmental impact and that contributes to the elimination of poverty, food and nutrition insecurity, and poor health outcomes (Johnston et al. 2014). The concept is similar to the Food and Agriculture Organization (FAO) definition of a climate-smart agriculture, a system that fights climate changes while consequently enhancing food security, as both are closely related (FAO 2010). It is part of a sustainable diet to reduce the consumption of meat: livestock production represents the largest emitter of methane as well as the largest user of land resources, causing land degradation and deforestation (FAO 2010). It is important to notice that the food products used to replace the meat should provide the same nutritional benefits, with less harm to the environment. The products that fit these demands are known as meat substitutes, meat analogues, meat replacers, or meat alternatives (Hoek et al. 2011) and they can be based on plant (e.g. soy, pea, and oat), animal (e.g. milk and insects) or microbial products (e.g. mycoprotein) (Smetana et al. 2015).

Socially, the consumption of meat has been justified by the so-called 4Ns—the belief that it is natural, normal, necessary, and/or nice (Piazza et al. 2015). However, ethical and environmental concerns have recently induced a rapid expansion of the meat substitute market (Godfray et al. 2018), which is predicted to have an annual turnover of $6 billion in 2022 (Ritchie et al. 2017). The main reason for the substitution of the meat in a consumer’s diet is the possible nutritional benefit it can bring. Several studies have reported that red meat consumption may increase mortality (Pan et al. 2012; Rohrmann et al. 2013; Snowdon et al. 1984). Yet, to remove the meat from the diet, the taste and variety of the options are also considered (Asgar et al. 2010). Elzerman et al. (2011) reported out that replacing meat in a non-vegetarian’s diet is easier when the meat substitute fits in a meal, compared to when it is tasted separately. Therefore, a meat alternative does not necessarily need to resemble meat in texture, taste, and flavor, but it needs to look like meat. In this context, the substitution of meat by mycoprotein is considered a more realistic scenario than the change to protein-rich plants because mycoprotein is more similar to meat, easing the consumers’ acculturation process (Raats 2007). A comparison of different meat and meat alternative options is presented in Table 1.

**Table 1:** An overview of the animal meat and meat alternative options based on environmental impact, health benefits, consumers’ acceptance and safety

Product	Environment	Health	Acceptance	Safety
Beef	Highly resource consuming. High global warming potential (Sonesson et al. 2017)	Consumption of beef has been associated with cancer. It contains highly bioavailable nutrients, including essential amino acids, heme iron, and vitamin B12 (Pereira and Vicente 2013)	Widely accepted. In some cultures, the consumption of beef is associated with the images of strength and power (Kumar et al. 2017)	Highly perishable, with numerous outbreaks regarding spoiled products being reported each year (Gul et al. 2016)
Poultry	Less environmental impacting than beef (Smetana et al. 2015)	Replacing beef with chicken has caused no changes in cholesterol or triglycerides levels in blood compared to beef (Scott et al., 1994). Good source of niacin and vitamin B6 but poor in iron (Pereira and Vicente 2013)	The second most consumed type of meat and the fastest growing meat sector. Absent of cultural barriers (Petracci and Berri 2017)	Highly perishable, with numerous outbreaks regarding spoiled products being reported each year (Gul et al. 2016)
Fish	Less environmental impacting than beef. Big variation in environmental impact depending on the species and the fishing method (Clune et al. 2017). Overfishing imposes a threat to the future of this resource (Emanuelsson et al. 2014)	Consumption of fish promotes healthy heart in the aging population. It contains high-quality protein, vitamins, and other essential nutrients. Risk of presence of environmental contaminants (Domingo 2016)	Global consumption of fish has been reported to be increasing (Claret et al. 2016)	Similar to other animal meat. Marine pollution, heavy metals, antibiotics, parasites, healthy animal feeding and healthiness are points to be considered (Claret et al. 2016)
Mycoprotein	GHG emissions comparable to pork and chicken. More efficient in land and water use (Smetana et al. 2015)	Mycoprotein presents low content of fat and high contents of protein and fiber (Denny et al. 2008; Smith et al. 2015). Positive effects on blood cholesterol concentration (Ruxton and McMillan 2010) and glycemic response (Bottin et al. 2016; Denny et al. 2008; Turnbull and Ward 1995) have been reported	Mycoprotein reproduces well the taste and the consistency of meat, increasing the acceptance of this meat substitute (Matassa et al. 2016)	Some allergic reactions have been reported (Jacobson and DePorter 2018)
Soy protein	Soy protein figures among the less impacting options in most LCA categories analyzed (Smetana et al. 2015)	Its nutrient content is comparable to meat, with lower chances of causing cardiovascular diseases. A good source of calcium and linoleic acid (Kumar et al. 2017)	Soy-based products are perceived as having bad taste in Western societies, but their health benefits positively influences consumers (Fenko et al. 2015)	Overall safe (Kucuk 2017). One of the eight foods with most documented cases of allergy in the USA (Meinlschmidt et al. 2016)
Gluten	High impact on agricultural land occupation; overall impact similar to dairy options (Smetana et al. 2015)	The gluten is poor in lysine and threonine, both essential amino acids (Wouters et al. 2016)	Products made of gluten have been present in the supermarkets for a long time (Wouters et al. 2016)	Celiac disease, a permanent intolerance of gluten proteins, occurs in nearly 1% of the population in many countries (Asgar et al. 2010). Also part of the eight foods responsible for most allergy cases in the USA (Ahsan et al. 2016)
Insect	The environmental impact caused by insects is similarly to soy (Smetana et al. 2015)	The characteristics vary between species. The presence of all essential amino acids and a good saturated/unsaturated fatty acid ratio have been reported to some species. The exoskeleton made of chitin/chitosan is also attractive (Huis 2013)	Insects are commonly consumed in tropical countries but rare in the Western culture (Huis 2013)	Microbial, chemical, toxicological, and allergenic risks should be considered when developing a novel insect-based food. The consumption of the African silkworm (*Anaphe venata*), for example, is related to ataxic syndrome cases in southwest Nigeria (Huis 2013)
Mushroom	Mushrooms cause a high environmental impact (per kg of protein) compared to other vegetarian options because of their low protein content (Jungbluth et al. 2016)	They are rich in dietary fibers, polyunsaturated fatty acids, and high-quality proteins. They can reduce the harmful low-density lipoproteins and enhances the beneficial high density lipoproteins in blood (Kumar et al. 2017)	Their flavor is more acceptable than beany flavor of textured soy protein. Health benefits and antitumor effect of mushroom increased their acceptance among consumers (Kumar et al. 2017). They contain umami tastants with flavor enhancing properties (Guinard et al. 2016)	Fresh mushrooms are highly perishable, and are subject to extremely rapid microbiologic and biochemical changes (Niksic et al. 2016). Rare cases of food allergy to mushrooms have been reported (Hegde et al. 2002). Accumulation of trace elements (e.g. As) should be observed (Mleczek et al. 2016)
Cultured meat	Cultured meat demands large amounts of energy, resulting in an environmental impact more than 4 times higher than chicken (Smetana et al. 2015)	The biochemical composition of meat might be altered to make it healthier, e.g. the content of poly-unsaturated fatty acids can be increased (Post 2012)	Public acceptance depends on an efficient production and resemblance to meat (Post 2012)	The quality of the substrates and other compounds used in the culture medium imposes more risks than microbial contamination (Bhat and Fayaz 2011)
Microalgae	Smetana et al. (2018) have associated the production of meat alternatives from microalgae to high energy consumption and low water footprint	They present an interesting nutritional protein value as well as high amounts of omega-3 fatty acids (Weinrich and Elshiewy 2019)	The taste of Spirulina has been described as not fitting a stand-alone item. The use of flavorings and seasonings needs further investigation (Grahl et al. 2018). The possibility of an organic and local production can positively affect the consumers’ choice towards algae-based alternatives (Weinrich and Elshiewy 2019)	Generally, species like *Spirulina* sp. and *Chlorella* sp. are not known to toxic metabolites. However, the location of the cultivation ponds can expose the algae to toxic elements (e.g. heavy metals, As), which will be absorbed by the algae (Rzymski et al. 2015)

Mycoprotein refers to the proteinaceous food obtained from filamentous fungal biomass which can be used for human consumption. Mushrooms and truffles, also belonging to the Fungi Kingdom, have been part of the diet of many cultures thanks to their pleasant taste (Boland et al. 2013). However, they are not considered good meat substitutes because of their low content of proteins. Rapid growth and high protein content, on the other hand, make filamentous fungi important potential sources of protein for food (Anupama and Ravindra 2000). These fungi have been consumed for many years by humans as components of fermented food, aiming to prolong the shelf-life, reduce the volume, shorten the cooking time, and improve the nutritive value of the food (Nout and Aidoo 2002). In Europe, *Penicillium roquefortii* and *Penicillium camembertii* are used in the production of blue (Roquefort, Gorgonzola) and soft-ripened (Camembert and Brie) cheese, respectively. In Asia, *Monascus purpureus* is used in the production of red yeast rice; *Aspergillus oryzae* ferments soybeans to produce hamanatto, miso and shoyu (Moore and Chiu 2001). Alternatively, the filamentous fungal biomass can be processed and used as food, that is, mycoprotein. Mycoprotein has been designated as GRAS—Generally Recognized As Safe—by the Food and Drug Administration (FDA) in the US since 2002 (Denny et al. 2008).

Presently, one company (Quorn^®^, Marlow Foods, UK) commercializes mycoprotein products in supermarkets of 19 countries (Marlow Foods Ltd 2019). The fungus *Fusarium venenatum* is grown in a defined medium, treated to have its RNA content reduced, and added egg albumen, color and flavor compounds to mimic the texture and aspect of meat (Wiebe 2002). Research on appearance, texture, and mouthfeel of mycoprotein is limited to that associated with the production of *Fusarium venenatum* mycelial biomass. The present production method is costly, what results in market prices for mycoprotein similar to those of meat (Ritchie et al. 2017). The reduction of the cost involved in the production is one of the challenges to encourage the consumption of mycoprotein. Public awareness of the health and environmental benefits can also contribute to popularize mycoprotein. The present review provides a current environmental and health perspective of mycoprotein and future research avenues to encourage its production and consumption.

### Environmental aspects

According to Siegrist and Hartmann (2019), the consumer’s behavior is influenced by both the knowledge about the nutritional value and the perception of the environmental impact caused by the food. Therefore, the first step to popularize the consumption of the meat substitutes is to increase the public knowledge about the environmental impact of their dietary habits. The literature about life cycle analyses (LCA) of mycoprotein is scarce. Yet, they agree that mycoprotein causes less impact to the environment than beef. Finnigan et al. (2010) used an LCA to compare Quorn^®^ mince with beef mince and determined that, considered the same weight, the meat alternative generates only 48% of the global warming potential the animal protein causes. Uncertainties about the required amounts of glucose and egg albumen in the formulation of the mycoprotein product can increase this value to 60%. This study limited the system boundaries from the production of the raw materials to the factory gate.

Smetana et al. (2015) compared the environmental impact of mycoprotein produced from sugar beet molasses to chicken, lab grown meat, and dairy-, insect-, gluten- and soy based options. The bases used for the comparison were the weight, the calorific energy value, and the content of digestible bulk protein of each product. This was the first study involving mycoprotein to evaluate local and regional impact categories such as acidification, eutrophication and land use. According to the authors, the production of 1 kg of mycoprotein and 1 kg of chicken meat has similar impacts; and they are only lower than cultured meat (a technology in its early stages of development). Almost half of the mycoprotein overall impact (45%) comes from the mycoprotein processing; other 25% is the result of the frying at consumer, and 21% is associated with the components used in its production (10% for the egg white and 11% for the nitrogen fertilizer needed to grow the crops used as fungal substrate).

In the same work, the global warming potential for the mycoprotein has been determined to be 5.55–6.15 kg CO_2eq_ per kg of fungal product. Comparatively, chicken and pork have a global warming potential of 2–4 and 4–6 kg CO_2eq_ per kg of meat, respectively. When comparing the environmental impact of mycoprotein considering the calorific energy value and the content of digestible proteins, mycoprotein performed poorly. Only the impact of the cultured meat was superior to the mycoprotein.

In another work, Smetana et al. (2018) have reported the energy, land and water used in the production of meat substitutes. Mycoprotein figures among the most efficient alternatives when considering land (< 2 m^2^a/kg; compared to 5–7 m^2^a for chicken and 7–8 m^2^a for pork) and water use (~ 500 L/kg). For the energy consumption category, mycoprotein was as efficient as dairy alternatives (15–20 kWh/kg), but less efficient than vegetables and insects (less than 10 kWh/kg and 5–15 kWh/kg, respectively).

The use of LCA as a tool to compare the protein sources is important to provide information about the environmental impact of the food to the consumers. However, the definition of a functional unity for comparison of the food products still needs validation. Parameters such as kg of product, kg of protein, and kg of protein corrected by its digestibility score have been studied (Sonesson et al. 2017). Additionally, when focusing the comparisons on the protein content, other nutrients are neglected. In a preliminary study, Jungbluth et al. (2016) have determined the impacts of a complete home-cooked meal prepared with meat alternatives based on the Swiss ecological scarcity method from 2013. The meals were planned to provide a good balance of different nutrients. The mycoprotein option performed the worst among the vegetarian options studied, but was better than the meat and fish options used for comparison.

Utilization of agro-industrial residues for the manufacturing of mycoprotein is another strategy that can be considered to decrease the environmental impact of this meat substitute. Lignocellulosic materials without pretreatment can be used by filamentous fungus for the production of mycoprotein in submerged culture as well as in solid-state fermentation (Satari and Karimi 2018). The challenge in this alternative is to find agro-industrial waste streams that have beneficial nutritional composition to guarantee an efficient production. Moreover, if such streams are currently used for animal feed, the environmental impact caused by the replacement of these materials in the animal production can lead to an increased environmental impact. The successful utilization of the agri-food waste in the production of mycoprotein could reduce the environmental impact to 2–4 kg CO_2eq_, the use of land to 0.5 m^2^a, and the consumption of water and energy to 250 L and 10 kWh, respectively, per kg of mycoprotein (Smetana et al. 2018). However, the implementation of this technology in an industrial scale needs to previously overcome remaining challenges such as regulatory and safety approval, scale up of production, and large-scale trials (Lee et al. 2015).

### Nutrition and health

The human diet has rapidly changed over the last decades and our food is often suboptimal (Popkin et al. 2012). For food planning purposes, an appropriate protein intake should be of approximately 15 E% (i.e., 15% of the total energy intake). This corresponds to about 1.1 g of proteins per kg of body weight and day. For the elderly (≥ 65 years), an appropriate goal should be 18 E%, i.e., 1.2 g of proteins per kg of body weight and day (Nordic Council of Ministers 2014). An inadequate intake of proteins can result in edemas, muscle weakness, and detrimental changes in hair and skin (Nordic Council of Ministers 2014). Malnutrition over an extended period of time can lead to Protein-Energy Malnutrition (PEM) and result in serious diseases, such as kwashiorkor and marasmus (Batool et al. 2015).

Dunlop et al. (2017) investigated the impact of the mycoprotein ingestion on healthy young men, in a dose–response manner, on acute postprandial hyperaminoacidaemia and hyperinsulinaemia. The results demonstrated that the bioavailability of all amino acids (including the essential ones) in the mycoprotein is similar to milk and better than the plant-based protein sources. Additionally, an amount of 60 g of mycoprotein was determined to give an optimal response regarding muscle protein synthesis (Dunlop et al. 2017). Further research is needed in order to determine the optimal dose for various populations.

Mycoprotein is a low-fat, high-protein and high-fiber food component. On the other hand, its high RNA content raises some concerns. The fungal biomass originally contains 10% (dry weight) of RNA. Comparatively, edible offals such as beef liver and heart contain approximately 2 and 0.6% of RNA, respectively. Muscles contain even less (Jonas et al. 2001). The consumption of excessive quantities of RNA can lead to an increased amount of uric acid in the body, being therefore a risk factor for gout (Denny et al. 2008; Jonas et al. 2001). During its production, the biomass of *Fusarium venenatum* is submitted to a heat treatment. By rapidly heating the fungal biomass (still in the broth) to temperatures above 68 °C, and keeping it for 20–45 min, the RNA content of the mycoprotein is reduced to less than 2%. The thermal treatment acts by degrading the RNA into monomers that diffuse out of the cells (Raats 2007).

The mycoprotein contains a little amount of sodium and is a good source of zinc, selenium and antioxidants (Denny et al. 2008; Smith et al. 2015); yet the levels of iron and vitamin B12 are low compared to those found in red meat (Denny et al. 2008). Whether or not iron or other substances can be added to the growth medium in order to produce an enriched mycoprotein, with increased bioavailability, is worthy of further study.

The fiber present in the mycoprotein is composed of two-thirds β-glucan and one-third chitin, creating a “fibrous chitin–glucan matrix” with low water solubility (88% insoluble) (Bottin et al. 2016). The chitin is a polymer formed by N-acetylglucosamine monomers not commonly present in the human diet. Some potential effects the chitin ingestion causes in the health include the relief of joint pain in osteoarthritis and the stimulation of beneficial bacteria in the colon (Sadler 2004). Moreover, the mycoprotein’s fibers appear to improve the glycemic profile in a not-completely understood mechanism (Bottin et al. 2016; Denny et al. 2008; Turnbull and Ward 1995). Bottin et al. (2016) studied the effect of the consumption of mycoprotein in overweight and obese volunteers. Three amounts of mycoprotein were tested (44, 88 and 132 g per meal) and their results were compared to chicken meals containing the same energetic values. The ingestion of a meal containing mycoprotein improved the insulin sensitivity and decreased the insulin concentrations. Turnbull and Ward (1995) have reported that, for healthy individuals, the ingestion of mycoprotein has substantial effects on both glycemic and insulinemic variables 60 min after the meal, compared to a milk and soy flour option. More recently, Dunlop et al. (2017) also compared mycoprotein to milk in health subjects and concluded that the mycoprotein caused a slower but longer hyperinsulinaemia, i.e., the level of insulin in the blood increased less but was sustained for a longer period (the insulin peak was observed 45 min after the mycoprotein meal but only 15 min after the milk meal).

The consumption of mycoprotein possibly lowers the total blood cholesterol and the greatest benefits have been observed in subjects with raised cholesterol levels at baseline (Ruxton and McMillan 2010). Although an optimal intake of mycoprotein was not determined, the results suggest there might be a dose-dependent relationship (Denny et al. 2008). Additionally, compared to other protein sources such as chicken, the mycoprotein presents advantages regarding satiety (Bottin et al. 2016; Williamson et al. 2006). This might be due to the protein content as well as the fiber content, since both proteins and fibers have the ability to increase the feeling of satiety (Paddon-Jones et al. 2008; Slavin and Green 2007). It is possible that a diet including mycoprotein may fight hunger, reduce energy intake and facilitate weight loss. There is a need of longer-term studies with large sample sizes in order to fully understand the potential role of mycoprotein in relation to health and non-communicable diseases. More research is also needed to establish an optimal dose of mycoprotein to boost health for men, women, children, and older adults.

## Conclusion

Human dietary habits have changed and deeply affected our health, environment and society. Climate-smart food systems can help to reduce the negative impacts of this sector. Accordingly, substitution of the meat by meat analogues can present beneficial results in both personal and societal aspects. Mycoprotein is an interesting source of good-quality proteins, with good acceptance among consumers, and proven positive impacts in cholesterol, sugar, and insulin blood levels. On the other hand, the high price of this meat substitute narrows its consumption to developed markets, and the raw materials used in the product formulation impose a high environmental impact compared to other vegetarian options. Hence, alternative production processes using agro-industrial residues as substrate and solid state fermentation have been the subject of investigation.

## References

[CR1] Ahsan N, Rao RSP, Gruppuso PA, Ramratnam B, Salomon AR (2016). Targeted proteomics: current status and future perspectives for quantification of food allergens. J Proteom.

[CR2] Anupama, Ravindra P (2000). Value-added food: single cell protein. Biotechnol Adv.

[CR3] Asgar MA, Fazilah A, Huda N, Bhat R, Karim AA (2010). Nonmeat protein alternatives as meat extenders and meat analogs. Compr Rev Food Sci Food Saf.

[CR4] Augustin MA, Riley M, Stockmann R, Bennett L, Kahl A, Lockett T, Osmond M, Sanguansri P, Stonehouse W, Zajac I, Cobiac L (2016). Role of food processing in food and nutrition security. Trends Food Sci Technol.

[CR5] Batool R, Butt MS, Sultan MT, Saeed F, Naz R (2015). Protein-energy malnutrition: a risk factor for various ailments. Crit Rev Food Sci Nutr.

[CR6] Bhat ZF, Fayaz H (2011). Prospectus of cultured meat—advancing meat alternatives. J Food Sci Technol.

[CR7] Boland MJ, Rae AN, Vereijken JM, Meuwissen MPM, Fischer ARH, van Boekel MAJS, Rutherfurd SM, Gruppen H, Moughan PJ, Hendriks WH (2013). The future supply of animal-derived protein for human consumption. Trends Food Sci Technol.

[CR8] Bottin JH, Swann JR, Cropp E, Chambers ES, Ford HE, Ghatei MA, Frost GS (2016). Mycoprotein reduces energy intake and postprandial insulin release without altering glucagon-like peptide-1 and peptide tyrosine-tyrosine concentrations in healthy overweight and obese adults: a randomised-controlled trial. Br J Nutr.

[CR9] Claret A, Guerrero L, Gartzia I, Garcia-Quiroga M, Ginés R (2016). Does information affect consumer liking of farmed and wild fish?. Aquaculture.

[CR10] Clune S, Crossin E, Verghese K (2017). Systematic review of greenhouse gas emissions for different fresh food categories. J Clean Prod.

[CR11] Denny A, Aisbitt B, Lunn J (2008). Mycoprotein and health nutrition. Bulletin.

[CR12] Domingo JL (2016). Nutrients and chemical pollutants in fish and shellfish balancing health benefits and risks of regular fish consumption. Crit Rev Food Sci Nutr.

[CR13] Dunlop MV, Kilroe SP, Bowtell JL, Finnigan TJA, Salmon DL, Wall BT (2017). Mycoprotein represents a bioavailable and insulinotropic non-animal-derived dietary protein source: a dose–response study. Br J Nutr.

[CR14] Dunne JA, Williams RJ, Martinez ND (2002). Network structure and biodiversity loss in food webs: robustness increases with connectance. Ecol Lett.

[CR15] Elzerman JE, Hoek AC, van Boekel MAJS, Luning PA (2011). Consumer acceptance and appropriateness of meat substitutes in a meal context. Food Qual Prefer.

[CR16] Emanuelsson A, Ziegler F, Pihl L, Sköld M, Sonesson U (2014). Accounting for overfishing in life cycle assessment: new impact categories for biotic resource use. Int J Life Cycle Assess.

[CR17] FAO (2010). ‘Climate-smart’agriculture: Policies, practices and financing for food security, adaptation and mitigation.

[CR18] Fenko A, Backhaus BW, van Hoof JJ (2015). The influence of product- and person-related factors on consumer hedonic responses to soy products. Food Qual Prefer.

[CR19] Finnigan T, Lemon M, Allan B, Paton I (2010). Mycoprotein, life cycle analysis and the food 2030 challenge. Asp Appl Biol.

[CR20] Godfray HCJ, Beddington JR, Crute IR, Haddad L, Lawrence D, Muir JF, Pretty J, Robinson S, Thomas SM, Toulmin C (2010). Food security: the challenge of feeding. Billion People Sci.

[CR21] Godfray HCJ, Aveyard P, Garnett T, Hall JW, Key TJ, Lorimer J, Pierrehumbert RT, Scarborough P, Springmann M, Jebb SA (2018). Meat consumption, health, and the environment. Science.

[CR22] Grahl S, Palanisamy M, Strack M, Meier-Dinkel L, Toepfl S, Mörlein D (2018). Towards more sustainable meat alternatives: how technical parameters affect the sensory properties of extrusion products derived from soy and algae. J Clean Prod.

[CR23] Guinard J-X, Myrdal Miller A, Mills K, Wong T, Lee SM, Sirimuangmoon C, Schaefer SE, Drescher G (2016). Consumer acceptance of dishes in which beef has been partially substituted with mushrooms and sodium has been reduced. Appetite.

[CR24] Gul K, Singh P, Wani AA (2016). Chapter 4—Safety of meat and poultry. Regulating Safety of Traditional and Ethnic Foods.

[CR25] Hegde VL, Das JR, Venkatesh YP (2002). Anaphylaxis caused by the ingestion of cultivated mushroom (*Agaricus bisporus*): identification of allergen as mannitol. Allergol Int.

[CR26] Hoek AC, Luning PA, Weijzen P, Engels W, Kok FJ, de Graaf C (2011). Replacement of meat by meat substitutes. A survey on person- and product-related factors in consumer acceptance. Appetite.

[CR27] Huis A (2013). Potential of insects as food and feed in assuring food security. Annu Rev Entomol.

[CR28] Jacobson MF, DePorter J (2018). Self-reported adverse reactions associated with mycoprotein (Quorn-brand) containing foods. Ann Allergy Asthma Immunol.

[CR29] Johnston JL, Fanzo JC, Cogill B (2014). Understanding sustainable diets: a descriptive analysis of the determinants and processes that influence diets and their impact on health. Food Secur Environ Sustain Adv Nutr.

[CR30] Jonas DA, Elmadfa I, Engel KH, Heller KJ, Kozianowski G, König A, Müller D, Narbonne JF, Wackernagel W, Kleiner J (2001). Safety considerations of DNA in food. Ann Nutr Metab.

[CR31] Jungbluth N, Eggenberger S, Nowack K, Keller R (2016) Life cycle assessment of meals based on vegetarian protein sources. In proceedings from: the 10th international conference on life cycle assessment of food (LCA Food 2016).

[CR32] Kucuk O (2017). Soy foods, isoflavones, and breast cancer. Cancer.

[CR33] Kumar P, Chatli MK, Mehta N, Singh P, Malav OP, Verma AK (2017). Meat analogues: health promising sustainable meat substitutes. Crit Rev Food Sci Nutr.

[CR34] Lee JZ, Logan A, Terry S, Spear JR (2015). Microbial response to single-cell protein production and brewery wastewater treatment. Microb Biotechnol.

[CR35] Marlow Foods Ltd. (2019). https://www.quorn.co.uk/recipes. Accessed 03 Apr 2019

[CR36] Matassa S, Boon N, Pikaar I, Verstraete W (2016). Microbial protein: future sustainable food supply route with low environmental footprint. Microb Biotechnol.

[CR37] Meinlschmidt P, Ueberham E, Lehmann J, Schweiggert-Weisz U, Eisner P (2016). Immunoreactivity, sensory and physicochemical properties of fermented soy protein isolate. Food Chem.

[CR38] Mleczek M, Niedzielski P, Siwulski M, Rzymski P, Gąsecka M, Goliński P, Kozak L, Kozubik T (2016). Importance of low substrate arsenic content in mushroom cultivation and safety of final food product. Eur Food Res Technol.

[CR39] Moore D, Chiu SW (2001) Fungal products as food. In Pointing, SB and Hyde, KD (eds) Bio-exploitation of filamentous fungi. Fungal Divers Res Ser 6:223–251.

[CR40] Niksic M, Klaus A, Argyropoulos D (2016). Chapter 22—Safety of foods based on mushrooms. Regulating safety of traditional and ethnic foods.

[CR41] Nordic Council of Ministers (2014) Nordic Nutrition Recomendations 2012—integrating nutrition and physical activity, Copenhagen 5(11):1

[CR42] Nout M. J. R., Aidoo K. E. (2002). Asian Fungal Fermented Food. Industrial Applications.

[CR43] Paddon-Jones D, Westman E, Mattes RD, Wolfe RR, Astrup A, Westerterp-Plantenga M (2008). Protein, weight management, and satiety. Am J Clin Nutr.

[CR44] Pan AP, Sun QMDS, Bernstein AMMDS, Schulze MBD, Manson JEMDD, Stampfer MJMDD, Willett WCMDD, Hu FBMDP (2012). Red meat consumption and mortality: results from 2 prospective cohort studies. Arch Int Med.

[CR45] Pereira PMCC, Vicente AFRB (2013). Meat nutritional composition and nutritive role in the human diet. Meat Sci.

[CR46] Petracci M, Berri C (2017). Poultry quality evaluation: quality attributes and consumer values.

[CR47] Piazza J, Ruby MB, Loughnan S, Luong M, Kulik J, Watkins HM, Seigerman M (2015). Rationalizing meat consumption. The 4Ns. Appetite.

[CR48] Popkin BM, Adair LS, Ng SW (2012). Global nutrition transition and the pandemic of obesity in developing countries. Nutr Rev.

[CR49] Post MJ (2012). Cultured meat from stem cells: challenges and prospects. Meat Sci.

[CR50] Raats J (2007) Meat (substitutes) comparing environmental impacts. A Case study comparing Quorn and pork, University of Groningen

[CR51] Ritchie H, Laird J, Ritchie D (2017). 3f bio: Halving the cost of mycoprotein through integrated fermentation processes. Ind Biotechnol.

[CR52] Rohrmann S, Overvad K, Bueno-de-Mesquita HB, Jakobsen MU, Egeberg R, Tjønneland A, Nailler L, Boutron-Ruault M-C, Clavel-Chapelon F, Krogh V, Palli D, Panico S, Tumino R, Ricceri F, Bergmann MM, Boeing H, Li K, Kaaks R, Khaw K-T, Wareham NJ, Crowe FL, Key TJ, Naska A, Trichopoulou A, Trichopoulos D, Leenders M, Peeters PHM, Engeset D, Parr CL, Skeie G, Jakszyn P, Sánchez M-J, Huerta JM, Redondo ML, Barricarte A, Amiano P, Drake I, Sonestedt E, Hallmans G, Johansson I, Fedirko V, Romieux I, Ferrari P, Norat T, Vergnaud AC, Riboli E, Linseisen J (2013). Meat consumption and mortality—results from the European Prospective Investigation into Cancer and Nutrition. BMC Med.

[CR53] Röös E, Sundberg C, Tidåker P, Strid I, Hansson P-A (2013). Can carbon footprint serve as an indicator of the environmental impact of meat production?. Ecol Ind.

[CR54] Ruxton CHS, McMillan B (2010). The impact of mycoprotein on blood cholesterol levels: a pilot study. Br Food J.

[CR55] Rzymski P, Niedzielski P, Kaczmarek N, Jurczak T, Klimaszyk P (2015). The multidisciplinary approach to safety and toxicity assessment of microalgae-based food supplements following clinical cases of poisoning. Harmful Algae.

[CR56] Sadler MJ (2004). Meat alternatives—market developments and health benefits. Trends Food Sci Technol.

[CR57] Satari B, Karimi K (2018). Mucoralean fungi for sustainable production of bioethanol and biologically active molecules. Appl Microbiol Biotechnol.

[CR58] Scott LW, Dunn JK, Pownall HJ, Brauchi DJ, McMann MC, Herd JA, Harris KB, Savell JW, Cross HR, Gotto AM (1994). Effects of beef and chicken consumption on plasma lipid levels in hypercholesterolemic men. JAMA Intern Med.

[CR59] Siegrist M, Hartmann C (2019). Impact of sustainability perception on consumption of organic meat and meat substitutes. Appetite.

[CR60] Slavin J, Green H (2007). Dietary fibre and satiety. Nutr Bull.

[CR61] Smetana S, Mathys A, Knoch A, Heinz V (2015). Meat alternatives: life cycle assessment of most known meat substitutes. Int J Life Cycle Assess.

[CR62] Smetana S, Aganovic K, Irmscher S, Heinz V (2018). Agri-food waste streams utilization for development of more sustainable food substitutes. Designing sustainable technologies, products and policies: from science to innovation.

[CR63] Smith H, Doyle S, Murphy R (2015). Filamentous fungi as a source of natural antioxidants. Food Chem.

[CR64] Snowdon DA, Phillips RL, Fraser GE (1984). Meat consumption and fatal ischemic heart disease. Prev Med.

[CR65] Sonesson U, Davis J, Flysjö A, Gustavsson J, Witthöft C (2017). Protein quality as functional unit—a methodological framework for inclusion in life cycle assessment of food. J Clean Prod.

[CR66] Steinfeld H, Gerber P, Wassenaar TD, Castel V, Rosales M, Rosales M, de Haan C (2006) Livestock's long shadow: environmental issues and options. Food & Agriculture Org

[CR67] Tscharntke T, Clough Y, Wanger TC, Jackson L, Motzke I, Perfecto I, Vandermeer J, Whitbread A (2012). Global food security, biodiversity conservation and the future of agricultural intensification. Biol Conserv.

[CR68] Turnbull WH, Ward T (1995). Mycoprotein reduces glycemia and insulinemia when taken with an oral-glucose-tolerance test. Am J Clin Nutr.

[CR69] Weinrich R, Elshiewy O (2019). Preference and willingness to pay for meat substitutes based on micro-algae. Appetite.

[CR70] Wiebe M (2002). Myco-protein from Fusarium venenatum: a well-established product for human consumption. Appl Microbiol Biotechnol.

[CR71] Williamson DA, Geiselman PJ, Lovejoy J, Greenway F, Volaufova J, Martin CK, Arnett C, Ortego L (2006). Effects of consuming mycoprotein, tofu or chicken upon subsequent eating behaviour, hunger and safety. Appetite.

[CR72] Wouters AG, Rombouts I, Lagrain B, Delcour JA (2016). Impact of casein and egg white proteins on the structure of wheat gluten-based protein-rich food. J Sci Food Agric.

